# A Structural and Functional Elucidation of the Rumen Microbiome Influenced by Various Diets and Microenvironments

**DOI:** 10.3389/fmicb.2017.01605

**Published:** 2017-08-24

**Authors:** Simon Deusch, Amélia Camarinha-Silva, Jürgen Conrad, Uwe Beifuss, Markus Rodehutscord, Jana Seifert

**Affiliations:** ^1^Department of Feed-Gut Microbiota Interaction, Institute of Animal Science, University of Hohenheim Stuttgart, Germany; ^2^Department of Bioorganic Chemistry, Institute of Chemistry, University of Hohenheim Stuttgart, Germany

**Keywords:** rumen microbiome, dietary impact, metaproteomics, LC-ESI-MS/MS, 16S rRNA gene, NMR, CAZy

## Abstract

The structure and function of the microbiome inhabiting the rumen are, amongst other factors, mainly shaped by the animal's feed intake. Describing the influence of different diets on the inherent community arrangement and associated metabolic activities of the most active ruminal fractions (bacteria and archaea) is of great interest for animal nutrition, biotechnology, and climatology. Samples were obtained from three fistulated Jersey cows rotationally fed with corn silage, grass silage or grass hay, each supplemented with a concentrate mixture. Samples were fractionated into ruminal fluid, particle-associated rumen liquid, and solid matter. DNA, proteins and metabolites were analyzed subsequently. DNA extracts were used for Illumina sequencing of the 16S rRNA gene and the metabolomes of rumen fluids were determined by 500 MHz-NMR spectroscopy. Tryptic peptides derived from protein extracts were measured by LC-ESI-MS/MS and spectra were processed by a two-step database search for quantitative metaproteome characterization. Data are available via ProteomeXchange with the identifier PXD006070. Protein- and DNA-based datasets revealed significant differences between sample fractions and diets and affirmed similar trends concerning shifts in phylogenetic composition. Ribosomal genes and proteins belonging to the phylum of Proteobacteria, particularly Succinivibrionaceae, exhibited a higher abundance in corn silage-based samples while fiber-degraders of the Lachnospiraceae family emerged in great quantities throughout the solid phase fractions. The analysis of 8163 quantified bacterial proteins revealed the presence of 166 carbohydrate active enzymes in varying abundance. Cellulosome affiliated proteins were less expressed in the grass silage, glycoside hydrolases appeared in slightest numbers in the corn silage. Most expressed glycoside hydrolases belonged to families 57 and 2. Enzymes analogous to ABC transporters for amino acids and monosaccharides were more abundant in the corn silage whereas oligosaccharide transporters showed a higher abundance in the fiber-rich diets. Proteins involved in carbon metabolism were detected in high numbers and identification of metabolites like short-chain fatty acids, methylamines and phenylpropionate by NMR enabled linkage between producers and products. This study forms a solid basis to retrieve deeper insight into the complex network of microbial adaptation in the rumen.

## Introduction

Ruminant livestock with about 3.6 billion farm animals globally (Hackmann and Spain, 2010) represents an important source of human food since these animals have the ability to convert plant-derived non-starch polysaccharides, indigestible for humans, to usable food products in form of milk and meat. This includes also an undesired side effect as ruminants release a substantial portion of methane, a potential greenhouse gas, to the atmosphere (McMichael et al., 2007). The underlying metabolic processes are driven by a complex microbial network consisting of archaea, bacteria, fungi, and protists residing in the strictly anaerobic rumen (Hungate, 1966; Mackie, 2000). Composition and activity of the rumen microbiome are, among other factors, primarily shaped by the diet (Ley et al., 2008; Henderson et al., 2015) and play an important role regarding the animals' health (Russell and Rychlik, 2001; Gressley et al., 2011) as well as feed efficiency and emission of environmentally harmful substances (Mizrahi, 2011; Shabat et al., 2016). Furthermore, the huge amount of fiber-degrading enzymes expressed in the rumen serves as a unique resource for the discovery of new lignocellulolytic enzymes useful for biofuel production (Brulc et al., 2009; Hess et al., 2011; Ferrer et al., 2012).

Bacteria, the most abundant (Krause and Russell, 1996; Mackie, 2000), diverse (McSweeney et al., 2005) and metabolically active species in the rumen are mainly responsible for the degradation and fermentation of plant fibers and proteins ingested by the animals (Hungate, 1966; Brulc et al., 2009). Bacterial species attached to feed particles constitute up to 75% of the total microbial population (McAllister et al., 1994; Koike et al., 2003). Others are free floating in the rumen fluid or live associated to fungi, protists, and the rumen epithelium (McAllister et al., 1994; Miron et al., 2001). Besides, bacteria can be classified according to their functional potential as there are, amongst others, fibrolytic, amylolytic, proteolytic, and saccharolytic species. Generally, starch and sugar degraders constitute the largest part of the ruminal bacterial population and are of great importance since diets for high-producing ruminants usually contain large amounts of readily fermentable starch and sugars. Despite their undoubtable significance, bacteria specialized for fiber degradation are typically less present (Puniya et al., 2015). Degradation of the entire organic matter taken in by the host animals cannot be achieved by a single organism but requires the functional capacities and cooperation of a succession of many microorganisms (Bladen et al., 1961). Hence, to obtain energy, bacterial communities interact synergistically in building diverse fibrolytic enzymes that finally yield in the production of short chain fatty acids and microbial protein which serve as the main energy and amino acid sources for the host (Hungate, 1966; Mackie, 2002).

Various methods have been employed to study the rumen microbiome ranging from classical cultivation (Bryant, 1959; Hungate et al., 1964) to molecular approaches including next generation sequencing (Edwards et al., 2004; Mackie and Cann, 2005; Creevey et al., 2014) and functional metagenomics (Brulc et al., 2009; Hess et al., 2011; Ferrer et al., 2012) as well as metabolomics (Ametaj et al., 2010; Saleem et al., 2013). Furthermore, investigations of diet induced shifts in microbial community composition of the rumen in different contexts are numerous (Tajima et al., 2001; Fernando et al., 2010; Kong et al., 2010; Pitta et al., 2010; de Menezes et al., 2011; Ann Huws et al., 2012; Belanche et al., 2012; Carberry et al., 2012; Petri et al., 2013; Thoetkiattikul et al., 2013; Zhang et al., 2013; Lengowski et al., 2016) but generally rumen studies are restricted to nucleic acids-based approaches with limited functional insights.

Bioinformatic and technical progress in mass spectrometry as well as a growing availability of reference sequences facilitate metaproteomic studies yielding increased information about taxonomic diversity, actual functional profiles, and interactions of the most active fractions of the investigated microbiota (Hettich et al., 2013; Seifert et al., 2013; Muth et al., 2016; Tanca et al., 2016). However, investigations of the prokaryotic rumen metaproteome are challenged by the complexity of rumen samples, which requires specific sample preparation procedures to separate archaeal and bacterial cells from the residual matter prior to protein extraction. Moreover, humic compounds are present and interfere with the metaproteomic workflow (Chourey et al., 2010; Heyer et al., 2013). Nevertheless, LC-ESI-MS/MS-based rumen studies have already been implemented successfully (Deusch and Seifert, 2015). The combination of different up to date *Omics*-technologies represents the most powerful tool to analyze the microbiome of complex ecosystems like the rumen (Lamendella et al., 2012; Deusch et al., 2015), but studies of the ruminal prokaryotic communities that include multiple approaches and state of the art methods are rare.

So far, to the best of our knowledge, there are no publications investigating the impact of the most common forages used as feed for dairy cows and fattening cattle on the metaproteome expressed by the entirety of archaeal and bacterial communities in the different phases of the rumen ecosystem. Complementary, structural and functional information obtained from the mass spectrometry-based analysis of the rumen metaproteome targeting the most metabolically active prokaryotes was further supplemented with Illumina MiSeq sequencing of the 16S rRNA gene that includes all cells present. Additionally, metabolome patterns were investigated by 500 MHz NMR spectroscopy. The aim of this investigation was to provide deeper insights into the complicated microbial network of the rumen ecosystem and its response to different animal diets to improve efficiency in animal production.

## Methods

### Ethics statement

The animals of this study were kept according to the German Animal Welfare legislation at the Agricultural Experiment Station Meiereihof of the University of Hohenheim in Stuttgart, Germany. The experimental procedures and treatments were authorized by the Regierungspräsidium Stuttgart in Germany as previously reported (Lengowski et al., 2016).

### Animals and diets

To access the dietary and host-related impact on the rumen microbiome a Latin square design using three rumen cannulated lactating Jersey cows was applied. Animals were fed rotationally with three different diets for *ad libitum* consumption and free access to drinking water. Feed was given once daily at 7.30 a.m. Based on dry matter, diets consisted of 52% concentrate mixture and 48% of either corn silage, grass silage, or grass hay. The concentrate was composed of 19% wheat, 19% barley, 7% soybean meal, 6% molasses, and 1% vitamin mineral premix. The corn silage-based diet was supplemented with urea to obtain a balanced nitrogen content in comparison to the grass silage- and hay-based diets. The chemical characteristics of the experimental diets are shown in Table 1.

**Table 1:** The measured chemical characteristics of the three forage sources and the thereof calculated properties of the final total mixed rations fed to the cows.

**Components (% of dry matter)**	**Basic forages**			**Total mixed rations**		
	**CS**	**GS**	**H**	**CS diet**	**GS diet**	**H diet**
Dry matter (%)	38.3	61.8	97.0	63.4	60.6	71.4
Crude ash	4.1	11.9	8.8	5.3	8.9	7.4
Ether extract	3.2	3.7	2.5	2.8	3.2	2.6
Crude protein	7.8	12.8	13.1	14.2	15.3	15.4
Neutral detergent fiber (organic)	41.3	52.9	58.9	27.2	33.2	36.0
Acid detergent fiber (organic)	21.0	31.8	33.6	13.3	18.8	19.6
Acid detergent lignin	1.7	2.6	3.0	1.0	1.4	1.6
Non-fiber carbohydrates	43.7	18.7	16.7	50.5	39.5	38.6

*CS, corn silage; GS, grass silage; H, grass hay*.

### Sampling

Samples were taken 5 h after feeding at 12.30 p.m. with a preceding adaptation time of 20 days for each diet. A quantity of 200 g of rumen matter was taken from five different positions (cranial, caudal, dorsal, ventral, medial) each and squeezed vigorously by hand using disposable polyethylene gloves to obtain the particle-associated liquid phase (LP) sample fraction, the remains constituted the solid phase (SP) sample fraction. Equal parts of the obtained LP and SP sample fractions were pooled across the five rumen positions. As a third sample fraction ventral rumen fluid (RF) was collected using a vacuum pump. Two times 27 samples (of three cows, diets and sample fractions) with 40 ml of the RF and LP sample fractions and 20 g of the SP sample fractions were frozen immediately at −80°C until further processing. The bacterial populations in the rumen can be subdivided into planktonic species, free-living in the RF and the fiber-adherent communities which can be further separated into groups of loosely and tightly attached species supposed to be present in the LP and the SP sample fractions, respectively (McAllister et al., 1994).

### Illumina amplicon sequencing

Using the FastDNA™ SPIN Kit for Soil (MP Biomedical, Solon, OH, USA) 250 mg of defrosted and vortexed samples were used for DNA extraction according to the manufacturer's instruction with slight modifications as described in Burbach et al. (2016). The extraction protocol included a bead-beating step for improved mechanical disruption of Gram-positive bacteria as suggested by Henderson et al. (2013). Quality and purity of DNA extracts were analyzed using a NanoDrop 2000 spectrophotometer (Thermo Fisher Scientific, Waltham, MA, USA). Illumina library preparation by PCR amplification of the V1-2 region of the 16S rRNA gene was performed as reported recently (Camarinha-Silva et al., 2014). The archaeal community was amplified using the previously described primers Arch349 and Arch806 (Lee et al., 2012). The forward primer contained a 6-nt barcode, a 2-nt linker and both primers comprised sequences complementary to the Illumina specific adaptors (Camarinha-Silva et al., 2014). The PCR mixture of a total volume of 20 μl contained PrimeSTAR HS DNA polymerase (2.5 U, Clontech Laboratories, Mountain View, CA, USA), 2.5 mM dNTP mixture, 0.2 μM primers and 1 μl of template DNA. An initial denaturation at 95°C for 3 min was followed by 20 cycles of denaturation at 98°C for 10 s, annealing at 59°C for 10 s, extension at 72°C for 45 s and a final extension for 2 min at 72°C. One microliter of the PCR product was used for a second PCR (15 cycles) under the same conditions with the reverse primer containing a sequence that integrated the Illumina multiplexing sequence and Illumina index primers (Camarinha-Silva et al., 2014). Integrity of amplicons was analyzed by gel electrophoresis, purified, and normalized using SequalPrep Normalization Kit (Invitrogen Inc., Carlsbad, CA, USA). Samples were pooled and sequenced using 250 bp paired-end sequencing chemistry on an Illumina MiSeq platform. Sequences were processed using the MOTHUR software pipeline (Kozich et al., 2013). Sequences were excluded if they had any primer or barcode mismatch or *N* character, aligned, checked for chimeras using UCHIME (Edgar et al., 2011) and clustered into operational taxonomic units (OTUs) at ≥97% similarity. Low abundance OTUs (<0.05% of total reads) were removed and a total of 1,484 bacterial and 626 archaeal phylotypes were taxonomically assigned using the naïve Bayesian RDP classifier (Wang et al., 2007) and the RDP database (Cole et al., 2014). Sequences were submitted to European Nucleotide Archive under the study accession number PRJEB19491. The mean number of sequence reads for bacteria and archaea was 41,410 ± 1,689 and 14,986 ± 1,713 respectively.

### Sample preparation for mass spectrometry

Samples were thawed and vortexed prior to sample preparation as described by Deusch and Seifert (2015). To detach firmly fiber-associated bacteria, 4 g of each SP sample were shaken horizontally for 2 h at 4°C in 35 ml precooled 50 mM Tris-HCl (pH 8; 0.2 M NaCl; 0.1% methylcellulose 400cP). To dilute the liquid fractions, 5 ml of the respective buffer were added to 8 g of the RF and LP samples. All samples were sonicated briefly for 1 min and pressed through two-layered sterile cheesecloth. Residues were rinsed again with 30 ml of the above mentioned buffer and pressed vigorously. Obtained filtrates were centrifuged at 200 × g for 10 min at 4°C and supernatants were further filtered through sterile 40 μm PE filters. Cells were pelleted at 10,000 × g for 15 min at 4°C and washed three times in 1 ml 50 mM Tris-HCl (pH 7.5; 0.1 mg/ml chloramphenicol; 1 mM PMSF). Subsequently, aliquoted cell pellets were stored at −20°C. Protein extraction was performed as described previously (Deusch and Seifert, 2015). Cell pellets were resuspended by vortexing in 200 μl 50 mM Tris-HCl (pH 7.5; 0.1 mg/ml chloramphenicol; 1 mM PMSF) and 300 μl of 20 mM Tris-HCl (pH 7.5; 2% SDS) were added. After shaking in a Thermo-Mixer (Eppendorf) for 10 min at 60°C and 1,200 rpm 1 ml of 20 mM Tris-HCl (pH 7.5; 0.1 mg/ml MgCl_2_; 1 mM PMSF; 1 μl/ml Benzonase, Novagen) was added. Cells were lysed by ultra-sonication on ice, four times 2 min (amplitude 60%; cycle 0.5) followed by shaking in a Thermo-Mixer for 10 min at 37°C and 1,200 rpm. Samples were centrifuged at 10,000 × g for 10 min at 4°C and proteins in the supernatant were precipitated for 30 min at 4°C using 20% precooled trichloroacetic acid. Subsequently, precipitates were centrifuged at 12,000 × g for 15 min at 4°C, protein pellets were washed twice in precooled acetone and dried by vacuum centrifugation. Protein pellets were resuspended in 35 μl Laemmli buffer by 5 min sonication bath and vortexing followed by incubation for 5 min at 95°C to reduce disulfide bonds. Twenty microliters were purified with a short run on a one-dimensional sodium dodecyl sulfate polyacrylamide gel electrophoresis (1D-SDS-PAGE; 4% stacking gel, 20 mA; 12% running gel, 40 mA). Each gel lane of 0.5 cm length representing one sample was cut out and subjected to in-gel trypsin (Promega, Madison, USA) digestion overnight (Jehmlich et al., 2008). Obtained peptides were purified and desalted using Stage tips equipped with five layers of Empore™ SPE Disks (C18; diameter 47 mm; thickness 0.5 mm) as described in detail by Rappsilber et al. (2007).

### LC-ESI-MS/MS measurements

LC-ESI-MS/MS analyses were performed in technical duplicates on an EasyLC 1000 nano-UHPLC (Thermo Scientific) coupled to a Q Exactive HF mass spectrometer (Thermo Scientific). Prior to LC-ESI-MS/MS measurements peptides were reconstituted in 20 μl 0.1% formic acid and 4 μl were injected by the autosampler. Separations of the peptide mixtures were done on a 20 cm fused silica emitter of 75 μm inner diameter (Proxeon Biosystems), in-house packed with reversed-phase ReproSil-Pur 120 C18-AQ 1.9 μm resin (Dr. Maisch GmbH). Peptide mixtures were injected onto the separation column in HPLC solvent A (0.1% formic acid) at a flow rate of 500 nl/min and eluted with a solvent B (80% acetonitrile in 0.1% formic acid) gradient of 1–33% within the first 73 min followed by an increase to 50% within 3 min plus an additional 3 min at 90%. The Q Exactive HF was operated in the positive ion mode. Full scan was acquired in the mass range from 300 to 1,650 m/z in the Orbitrap mass analyzer at a resolution of *r* = 120,000 followed by higher energy collisional dissociation (HCD) fragmentation of the twelve most intense precursor ions. High resolution MS/MS spectra were acquired with a resolution of *r* = 30,000. The target values were 3 × 10^6^ charges for the MS scans and 1 × 10^5^ charges for the MS/MS scans with a maximum fill time of 25 and 45 ms, respectively. The dynamic precursor exclusion was set to 30 s and peptide match was enabled.

### Bioinformatic data analysis

To improve the false discovery rate of peptide identifications and enhance the confidence of protein identifications, a two-step search approach was applied to create an artificial metagenome (Jagtap et al., 2013; Hansen et al., 2014). Therewith, the size of the search databases was reduced and simultaneously the sample-specificity was increased. First, all 54 raw data files were processed separately by Thermo Proteome Discoverer software (v. 1.4.1.14), Mascot engine (v. 2.4) in searching independently against the UniProtKB/TrEMBL databases (v. April 28, 2016) for bacteria (Taxonomy ID 2; 40,026,301 sequences) and archaea (Taxonomy ID: 2157; 1,200,545 sequences). Oxidation of methionine was set as variable modification and carbamidomethylation of cysteine as fixed modification. Precursor ion tolerance was defined at 10 ppm and fragment ion tolerance at 0.02 Da with two missed trypsin cleavages. Furthermore, all peaks besides the top 12 peaks per 100 Da in each MS/MS were removed to denoise spectra before identification and the Percolator node was activated with a false discovery rate of 1%. Using Thermo Proteome Discoverer, protein grouping was enabled with a minimum PSM confidence of medium and a delta Cn better than 0.15, strict maximum parsimony principle was applied. As a second step, the protein identifications inferred from the previous process were used to create sample-specific databases for label-free quantification (LFQ) of proteins via MaxQuant (v. 1.5.3.8) as previously demonstrated (Cox et al., 2014). The final in-house databases contained 22,331 bacterial and 818 archaeal protein sequences. The LFQ modality of MaxQuant was enabled with a minimum ratio count of two. Matching between runs with a match time window of 0.7 min and re-quantification was applied. Technical duplicates were combined to one experiment. Oxidation of methionine was set as variable modification with a maximum of five modifications per peptide and carbamidomethylation of cysteine was set as fixed modification. Besides, the default settings of MaxQuant were kept which included two missed trypsin cleavages, fully tryptic peptides, a peptide and protein false discovery rate below 1%, at least one peptide per protein (Gupta and Pevzner, 2009), a precursor mass tolerance of 4.5 ppm after mass recalibration and a fragment ion mass tolerance of 20 ppm. The phylogenetic composition was inferred from the proteins quantified by MaxQuant as annotated in the UniProtKB/TrEMBL database. KEGG Orthology (KO) identifiers and Cluster of Orthologous Groups of proteins (COG) were assigned using WebMGA (Wu et al., 2011) with an *e*-value cutoff of 10^−3^ considering exclusively the best hits. In order to link the quantified proteins to pathway maps of carbohydrate metabolism, KO identifiers were translated and grouped manually to the respective KEGG REACTION numbers as defined by the KO database. Carbohydrate-active enzymes (CAZymes) were annotated by searching the quantified bacterial proteins against the database for automated CAZyme annotation with hidden Markov models (dbCAN HMMs v. 5.0, based on the CAZyDB v. July 15, 2016) using hmmscan of the HMMER3 software package (Yin et al., 2012) and considering entirely the best *e*-value hits. The mass spectrometry data have been deposited to the ProteomeXchange Consortium via the PRIDE partner repository (Vizcaino et al., 2016) with the dataset identifier PXD006070.

### Nuclear magnetic resonance spectroscopy

The RF samples were defrosted, vortexed, and filtered through sterile 100 μm PE filters. Filtrates were centrifuged at 13,000 × g for 30 min at 4°C. Obtained supernatants were sterilized by passing through a 0.22 μm syringe filter and 3 ml of each RF sample were dried completely by vacuum centrifugation at room temperature overnight. By vigorous vortexing and 5 min brief sonication, dehydrated RF samples were reconstituted in 1.5 ml 50 mM sodium phosphate buffer (pH 6.5) in 99.9% deuterium oxide (Sigma-Aldrich, Germany) containing 5 mM 3-(trimethylsilyl)propionic-2,2,3,3-d_4_ acid sodium salt (TSP; Sigma-Aldrich, Germany) as an internal chemical shift reference and quantification standard. Subsequently, dissolved samples were centrifuged at 13,000 × g for 30 min at 4°C and 1 ml supernatant was transferred to a 5 mm glass NMR tube for measuring at 500 MHz using a Varian INOVA NMR spectrometer (Agilent Technologies). All ^1^H-NMR spectra were acquired at 25°C using the first transient of the noesy presaturation pulse sequence (Saude et al., 2006). Each spectrum was collected with 32 transients using a 4 s acquisition time, 1 s recycle delay and a mixing time of 0.1 s at a spectral width of 6,490 Hz. Spectral assignments were performed by 2D homonuclear and heteronuclear NMR: DQFCOSY, gHSQCAD, gHSQCTOCSY, as well as gHMBCAD were run using CHEMPACK 7.2 pulse sequences implemented in VnmrJ 4.2 (Agilent Technologies Inc., Santa Clara, CA, USA). Additionally, ^1^H-NMR spectra were imported into the Chenomx NMR Suite 8.2 software (database available at pH 6.5, Chenomx Inc., Edmonton, AB, Canada) for quantification (Weljie et al., 2006; Wishart, 2008) as described in Ametaj et al. (2010). Spectra were referenced to TSP (δ 0.0 ppm) for chemical shift and quantification. Prior to spectral analysis, all free induction decays (FIDs) were automatically zero-filled to 64 k data points, corrected for phase and baseline distortions and a line broadening of 0.5 Hz was applied. Concentrations of identified metabolites were divided by a factor of two since dried RF samples were reconstituted in half of the initial volume.

### Statistical analyses

The LFQ abundance values of proteins and the OTU counts were analyzed using the Primer 6 (v. 6.1.16) and Permanova+ (v. 1.0.6) statistical software package (PRIMER-E, Plymouth, UK). Non-metric multidimensional scaling (NMDS) was performed using the Bray–Curtis similarity matrix (Bray and Curtis, 1957). One-way analysis of similarities (ANOSIM) was used to determine statistical differences in protein and OTU abundance between diets, sample fractions and host animals (Clarke and Warwick, 2001). One-way analysis of variance (ANOVA) with *post-hoc* Tukey HSD (Honestly Significant Difference) was used for pairwise comparisons of the abundance means of taxonomic groups in diets and sample fractions and to test differences of metabolite concentrations in diets (IBM SPSS Statistics, Version 20.0. Armonk, NY: IBM Corp).

## Results

### Metaproteomics and amplicon sequencing of the rumen microbiome

**Figure 1:**
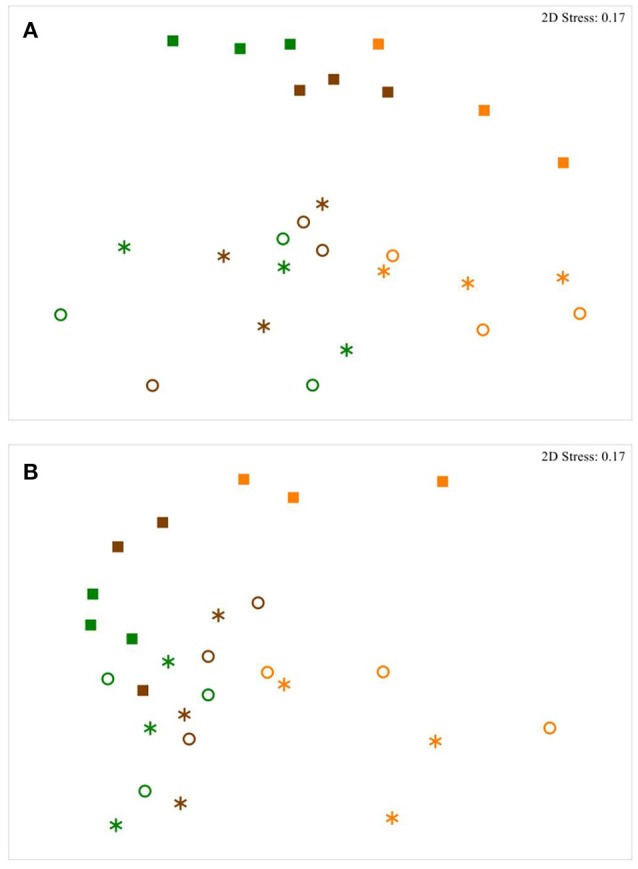
Non-metric multidimensional scaling (NMDS) plots of the metaproteome **(A)** and the bacterial community structure **(B)**. Yellow, corn silage-based diet; green, grass silage-based diet; brown, grass hay-based diet. Squares, solid phase; stars, liquid phase; circles, rumen fluid.

Over all samples 8,163 bacterial and 358 archaeal proteins were quantified by mass spectrometric measurements of the peptides and a two-step search identification and quantification process. Illumina MiSeq sequencing of the V1-2 region of 16S rRNA gene resulted in 1,484 bacterial and 626 archaeal OTUs assigned (Table S1). Regarding the abundances of bacterial proteins and OTUs, NMDS plots based on the Bray Curtis similarity revealed diet induced shifts and variations between sample fractions with similar trends for both, the metaproteomic dataset (Figure 1A) and the DNA-based approach (Figure 1B). There were no dietary, sample fraction or host related effects on the abundance of archaeal proteins and OTUs. ANOSIM verified significant differences in bacterial protein abundances regarding the diets with an *R*-value of 0.600 (*P* = 0.0001) for the corn silage- and grass silage-based samples and 0.442 (*P* = 0.0001) for the corn silage- and hay-derived samples. Respectively, the numbers of assigned bacterial OTUs of the corn silage- and grass silage-based samples differed with an *R*-value of 0.500 (*P* = 0.0003) and the corn silage- and hay-derived samples showed an *R*-value of 0.558 (*P* = 0.0001). Furthermore, ANOSIM confirmed significant variations in protein abundances between sample fractions with an *R*-value of 0.362 (*P* = 0.0001) for the LP and SP fractions. The RF and SP fractions differed with an *R*-value of 0.321 (*P* = 0.0008). Concerning the sequencing data, the *R*-value for the LP and SP fractions was 0.596 (*P* = 0.0001) and 0.561 (*P* = 0.0002) for the RF and SP fractions. Complete statistical information of the bacterial protein and OTU abundances considering diets, sample fractions and the individual cows is shown in Table 2.

**Table 2:** Analysis of similarity of the metaproteomic- and the 16S rRNA gene-based datasets.

		**LFQ-values of 8,163 bacterial proteins**		**Abundance of 1,484 bacterial OTUs**	
		***R***	***P***	***R***	***P***
	Diets^*^	0.418	0.0001	0.366	0.0001
	Fractions^*^	0.244	0.0004	0.379	0.0001
	Cows	0.143	0.0090	0.176	0.0070
Diets	GS:H	0.248	0.0100	0.078	0.1630
	CS:GS^*^	0.600	0.0001	0.500	0.0003
	CS:H^*^	0.442	0.0001	0.558	0.0001
Fractions	RF:LP	0.053	0.1870	−0.045	0.6980
	RF:SP^*^	0.321	0.0008	0.561	0.0002
	LP:SP^*^	0.362	0.0001	0.596	0.0001
Cows	28:59	0.088	0.1090	0.133	0.0790
	23:59	0.169	0.0280	0.207	0.0280
	23:28	0.180	0.0150	0.202	0.0270

The upper part of the table shows the global R statistics and the respective probability values (P) for the main factors (diets, sample fractions and individual cows). The lower part shows the R statistics and corresponding probability values (P) for the pairwise group comparisons within the main factors. Significantly different main factors and groups within are marked (^*^). CS, corn silage-based diet; GS, grass silage-based diet; H, hay-based diet; RF, rumen fluid; LP, liquid phase; SP, solid phase; cows are indicated by no. 23, 28, and 59.

### Dietary impact on community structure and variations in fractions

Taxonomic information was obtained from quantified proteins as annotated by the UniProtKB/TrEMBL database and from RDP retrieved OTU assignments of the Illumina amplicon sequencing. The taxonomic distribution of archaeal and bacterial proteins and OTUs in each sample at phyla, class, order, and family level as well as the corresponding numbers of proteins and OTUs are listed in Table S2. Overall, the bacterial community composition was dominated by the phylum of Bacteroidetes followed by the phylum of Firmicutes. Less abundant phyla were Actinobacteria, Elusimicrobia, Proteobacteria, Spirochaetes, Synergistetes, Tenericutes, and Verrucomicrobia (Table S2).

**Figure 2:**
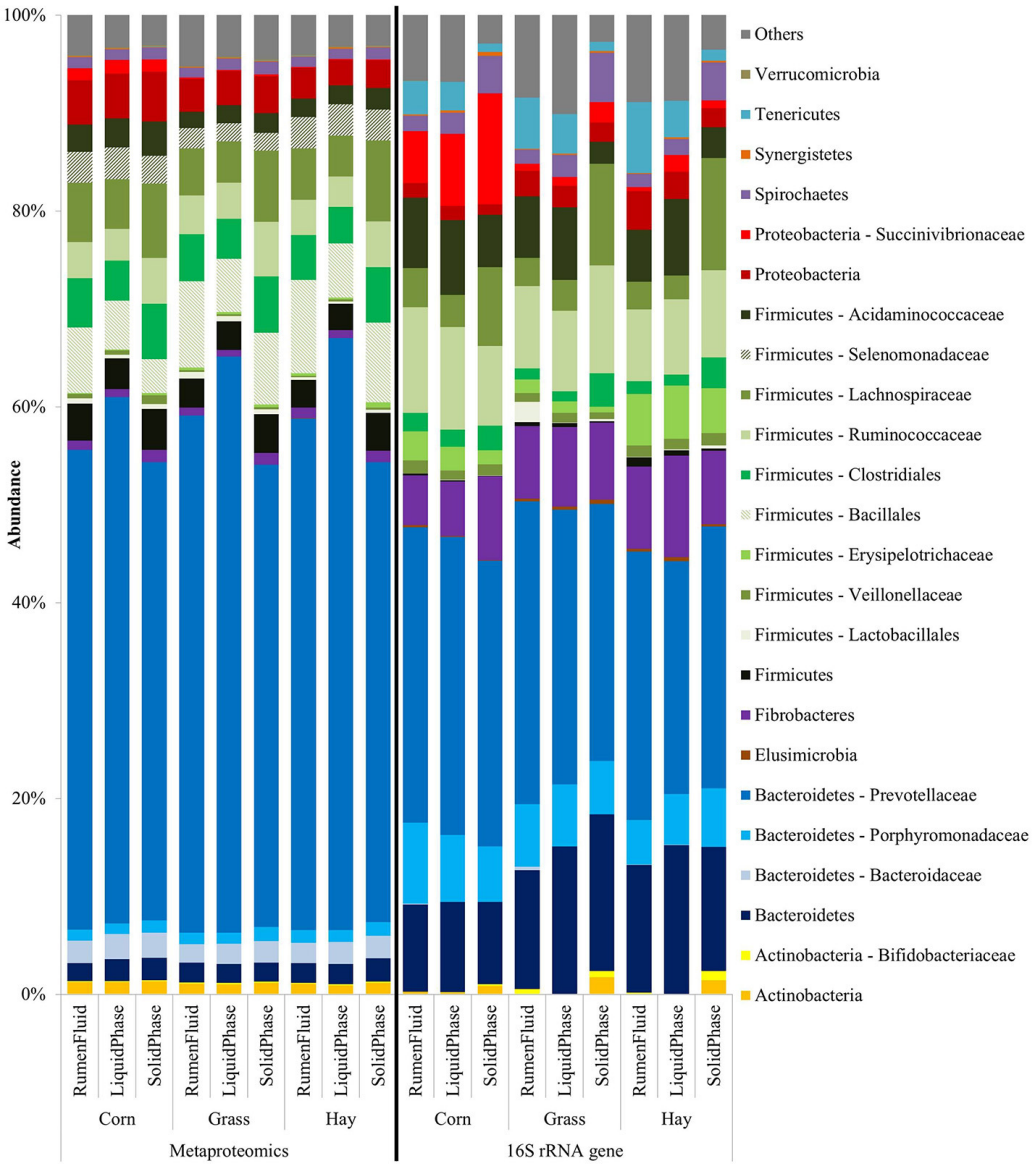
Bacterial phylogenetic distribution in diets and sample fractions (*n* = 3) obtained from the metaproteome (8,163 proteins) on the left and the 16S rRNA gene sequencing (1,484 OTUs) on the right. The order of Bacillales and the family of Selenomonadaceae were found exclusively in the metaproteomic dataset (shaded coloring).

However, the bacterial community structure averaged over the three animals per treatment revealed concordant tendencies concerning the dietary influence and variations in different microenvironments for both applied methods. Figure 2 shows the average abundance in diets and sample fractions of bacterial phyla, orders and families commonly identified by metaproteomics on the left and 16S rRNA gene sequencing on the right side. Proteins and OTUs belonging to the phylum of Proteobacteria including the family of Succinivibrionaceae were significantly more abundant throughout the samples of the corn silage-based diet when compared to the grass silage- and hay-based samples with *P* < 0.01 (LFQs) and *P* < 0.05 (OTUs). Within the corn silage-derived samples the respective proteins and OTUs were more abundant in the SP fraction (Figure 2). The abundance of OTUs assigned to Succinivibrionaceae was higher when compared to the LFQ-values of the corresponding proteins. Likewise, the family of Acidaminococcaceae belonging to the phylum of Firmicutes showed a higher protein (*P* < 0.01) and OTU abundance in the corn silage-based diet when compared to the grass silage- and hay-based samples (Figure 2). The abundance of the respective OTUs was least in the SP fraction of all diets whereas the LFQ-values of the corresponding proteins were highest in the SP fractions (Figure 2). The Firmicutes family of Selenomonadaceae was identified exclusively in the protein-based dataset (Figure 2). Selenomonadaceae proteins exhibited a lower abundance in the grass silage-derived sample fractions when compared to the corn silage- and hay-based samples (*P* < 0.01). Members of the order of Clostridiales including the families of Lachnospiraceae and Ruminococcaceae constituted major parts of the Firmicutes phylum (Figure 2) and revealed the highest LFQ-values and numbers of OTUs in the SP fractions of all diets when compared to the respective RF and LP fractions with *P* < 0.01 (LFQs) and *P* < 0.05 (OTUs). No OTUs were assigned to the Firmicutes order of Bacillales although highly abundant proteins were identified by mass spectrometry. Within diets, Bacillales proteins were more abundant in the RF fractions. The Erysipelotrichaceae family was more abundant in the hay-based diet when compared to the corn silage- and grass silage-based samples (Figure 2) emphasized primarily by the abundance of assigned OTUs (*P* < 0.01). Proteins and OTUs belonging to the order of Lactobacillales and to the family of Veillonellaceae constituted only a small part of the Firmicutes phylum as determined by both approaches (Table S2). Proteins of the Veillonellaceae family were more abundant in the corn silage-based diet when compared to the grass silage- and hay-based samples (*P* < 0.05). The abundance of OTUs belonging to the phylum of Fibrobacteres was higher when compared to the LFQ-values of the corresponding proteins (Figure 2). According to the 16S rRNA gene sequencing, the phylum of Fibrobacteres was less abundant in the RF and LP fractions of the corn silage-based diet when compared to the respective fractions of the grass silage- and hay-based diets. Within diets, the abundance of Fibrobacteres proteins was higher in the SP fractions of all diets when compared to the RF and LP samples (*P* < 0.05). Related to the overall bacterial community structure, OTU and LFQ-values assigned to the phylum of Elusimicrobia exhibited a low abundance across all samples (Table S2). Regardless the dietary treatments, the Prevotellaceae family dominated the phylum of Bacteroidetes and the overall bacterial community composition as determined by the protein- and DNA-based approaches (Figure 2). Within diets, Prevotellaceae proteins showed the highest LFQ-values in the LP fractions (*P* < 0.05). The family of Porphyromonadaceae exhibited a higher abundance of OTUs when compared to the LFQ-values of the respective proteins. In the same way, sequences assigned to the phylum of Bacteroidetes were more abundant than the LFQ-values of the corresponding proteins (Figure 2). In contrast, the family of Bacteroidaceae showed a higher abundance of proteins when compared to the abundance of the corresponding OTUs (Figure 2). OTU and LFQ-values of Bifidobacteriaceae were higher in the grass silage- and hay-based samples when compared to the corn silage-based fractions (Table S2). The abundance of OTUs and LFQs of the Actinobacteria phylum including the family of Bifidobacteriaceae was higher in the SP fractions of all diets when compared to the respective RF and LP fractions (Figure 2). Within diets, the phylum of Spirochaetes exhibited the highest abundance in the SP fractions as found by both methods. Similarly, the phylum of Synergistetes revealed the highest abundance of proteins and particularly OTUs in the SP fractions when compared to respective the RF and LP fractions (Figure 2). Proteins assigned to the phylum of Tenericutes were low abundant when compared to the corresponding OTU abundance. OTUs of the phylum of Tenericutes were more abundant in the RF and LP fractions when compared to the respective SP sample fractions whereas the LFQ-values of the corresponding proteins were higher in the SP fractions of all diets (Figure 2). In contrast, proteins of the Verrucomicrobia phylum were more abundant in comparison to the respective OTUs (Table S2).

**Figure 3:**
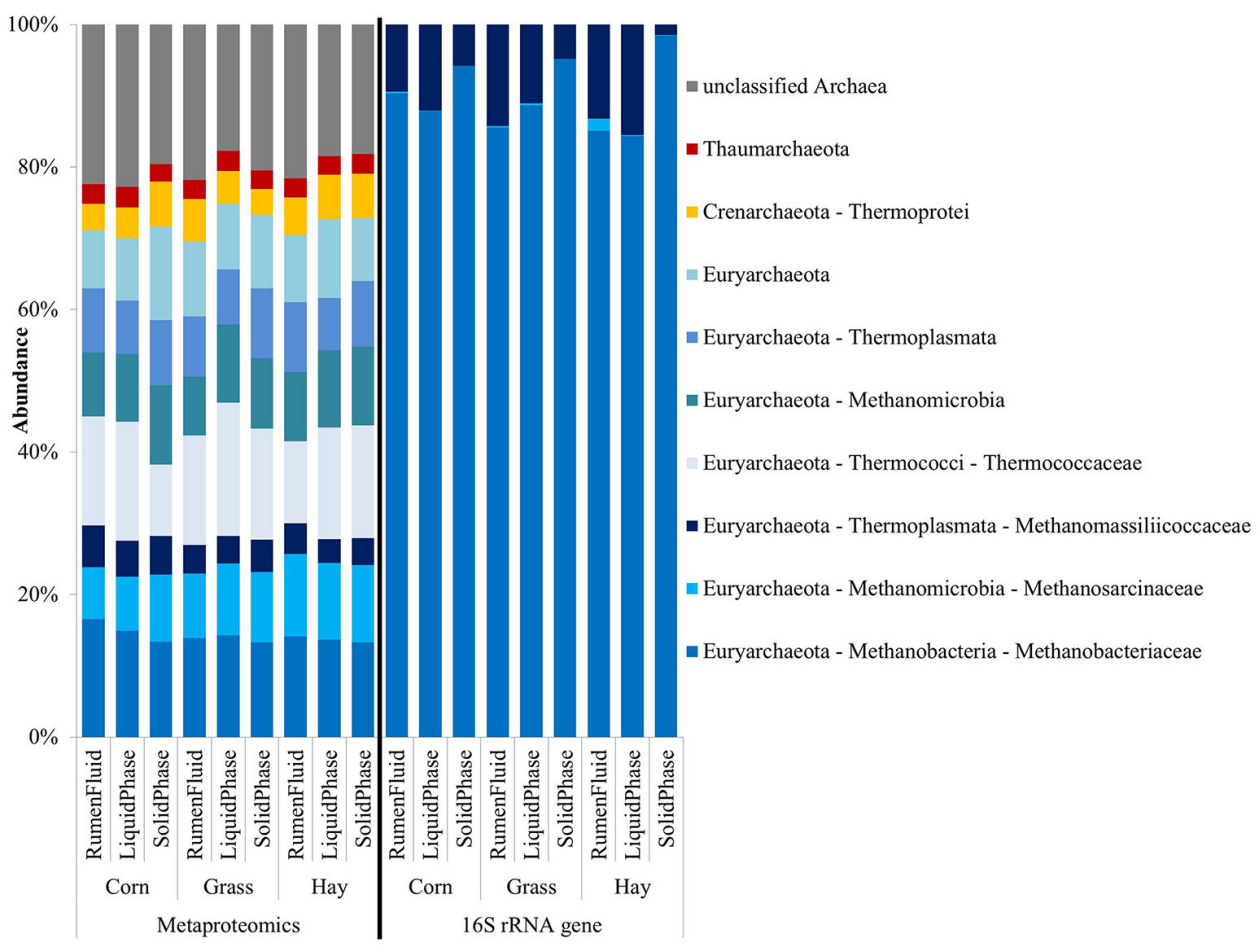
Archaeal phylogenetic distribution in diets and sample fractions (*n* = 3) obtained from the metaproteome (358 proteins) on the left and the 16S rRNA gene sequencing (626 OTUs) on the right.

Figure 3 shows the average abundance in diets and sample fractions of archaeal phyla, classes and families identified by metaproteomics on the left and 16S rRNA gene sequencing on the right. The metaproteomics-based approach identified 358 archaeal proteins that distributed over all archaeal phyla including as well the family of Thermococcaceae whereas the 626 OTUs were exclusively assigned to three families of methanogens: Methanobacteriaceae, Methanosarcinaceae, and Methanomassiliicoccaceae (Figure 3). Proteins belonging to the phyla of Thaumarchaeota and Crenarchaeota constituted a minor part of the total archaeal protein abundance (Figure 3). The LFQ-values of unclassified archaeal proteins were above 16% in each sample (Table S2). Within the phylum of Euryarchaeota, proteins belonging to the families of Methanobacteriaceae, Methanosarcinaceae, Thermococcaceae, and the class of Methanomicrobia were most abundant while OTUs of the family of Methanobacteriaceae prevailed with above 72% in each sample (Table S2).

### Carbohydrate-active enzymes

**Figure 4:**
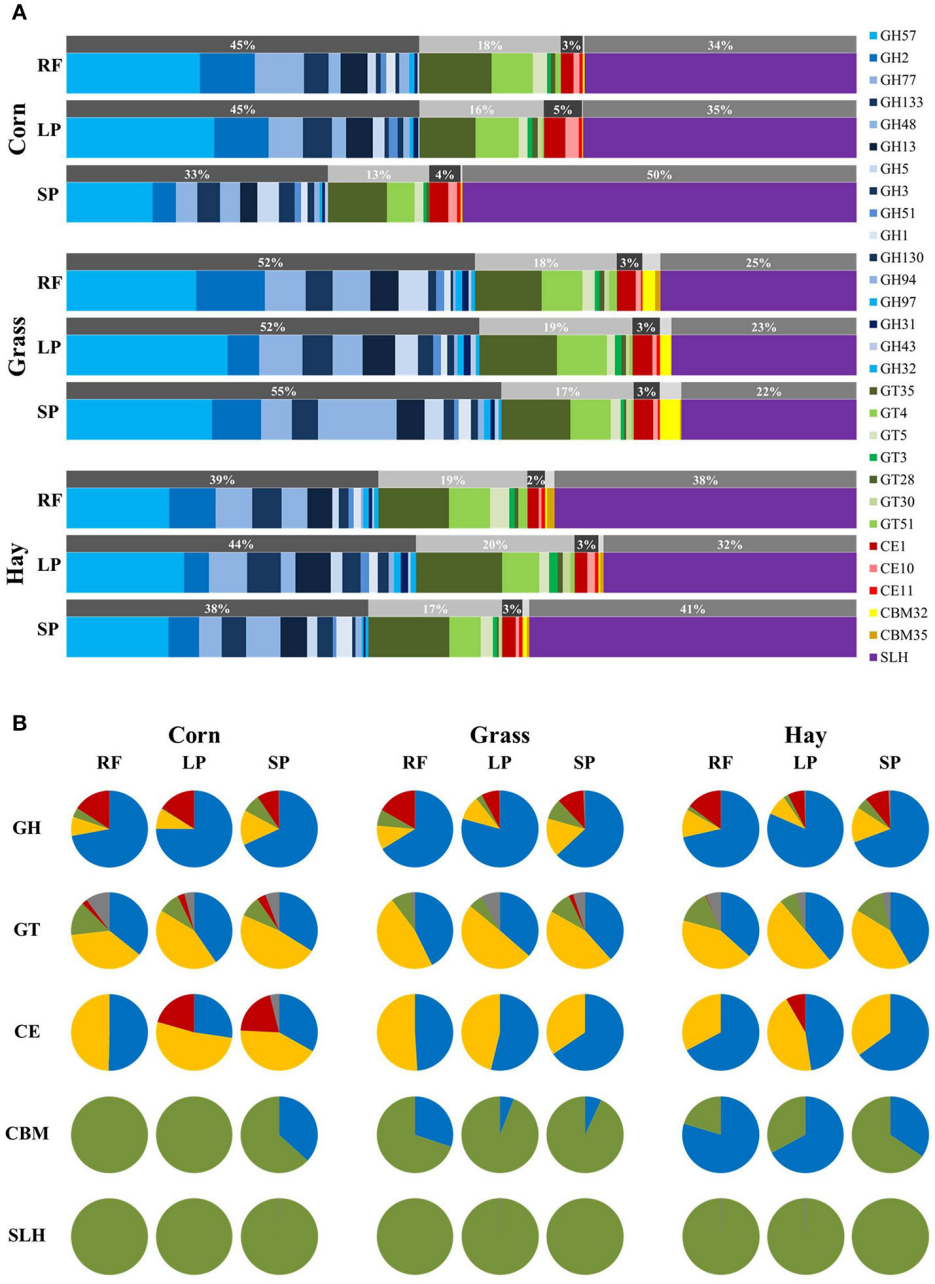
Abundance of 166 CAZymes in diets and sample fractions (*n* = 3). Glycoside hydrolases = GH (91 proteins), glycosyltransferases = GT (38 proteins), carbohydrate esterases = CE (16 proteins), carbohydrate-binding modules = CBM (2 proteins) and S-layer homology domains = SLH (19 proteins) **(A)**. Pie charts depict the phylogenetic origin of CAZymes of the respective categories in diets and sample fractions at phyla level: blue, Bacteroidetes; green, Firmicutes; red, Proteobacteria; yellow, environmental samples; gray, others **(B)**. RF, rumen fluid; LP, liquid phase; SP, solid phase.

CAZyme annotation with hidden Markov models identified a total of 166 bacterial proteins in five CAZy-categories (Table S3). A majority of 91 proteins were assigned to 16 glycoside hydrolase (GH) families and 38 proteins belonged to seven glycosyltransferase (GT) families. Furthermore, 16 proteins were assigned to three families of carbohydrate esterases (CE) and two proteins fell into two families of carbohydrate-binding modules (CBM). In addition, 19 proteins with sequence similarity to S-layer homology domains (SLH) were present in high abundance throughout diets and sample fractions. The percentage abundance of the respective bacterial CAZymes in diets and sample fractions in relation to the total abundance of CAZymes is shown in Figure 4A. The phylogenetic origin at phyla level of the five CAZy-categories in diets and sample fractions is depicted in Figure 4B. The ratio of the most abundant CAZy-categories of GH, mainly produced by Bacteroidetes species and SLH, almost exclusively derived from Firmicutes species varied between the diets (Figures 4A,B). Proteins of the SLH category were less abundant in the grass silage-based sample fractions when compared to the corn silage- and hay-based samples. Contrarily, proteins of the GH category were more abundant in the grass silage-based diet in comparison to the corn silage- and hay-based samples (Figure 4A). Proteobacteria-derived proteins belonging to the GT category were more abundant in the corn silage-based samples when compared to grass silage- and hay-based samples (Figure 4B). Proteins assigned to the CE category were more abundant in the LP and SP fractions of the corn silage-based diet when compared to the respective fractions of the grass silage and hay-based diets (Figure 4A). Moreover, the LP and SP fractions of the corn silage-based diet exhibited a higher abundance of proteobacterial proteins in the CE category when compared to the remaining samples (Figure 4B). CBM related proteins were more abundant in the sample fractions of the grass silage-based diet when compared to the corn silage- and hay-based diets (Figure 4A). Across all samples, the family GH57, based on seven proteins like alpha-amylases, was most abundant (Figure 4A). The most expressed GT family was GT35 including 18 proteins of different glycogen and starch phosphorylases. Within the category of CE the most expressed family was CE1 with eight proteins. The CBM32 family was most abundant in the SP fraction of the grass silage-based diet (Figure 4A). Details about the proteins assigned to the different CAZy-categories including the phylogenetic origin and the respective functions are listed in Table S3.

### ABC transporters

**Figure 5:**
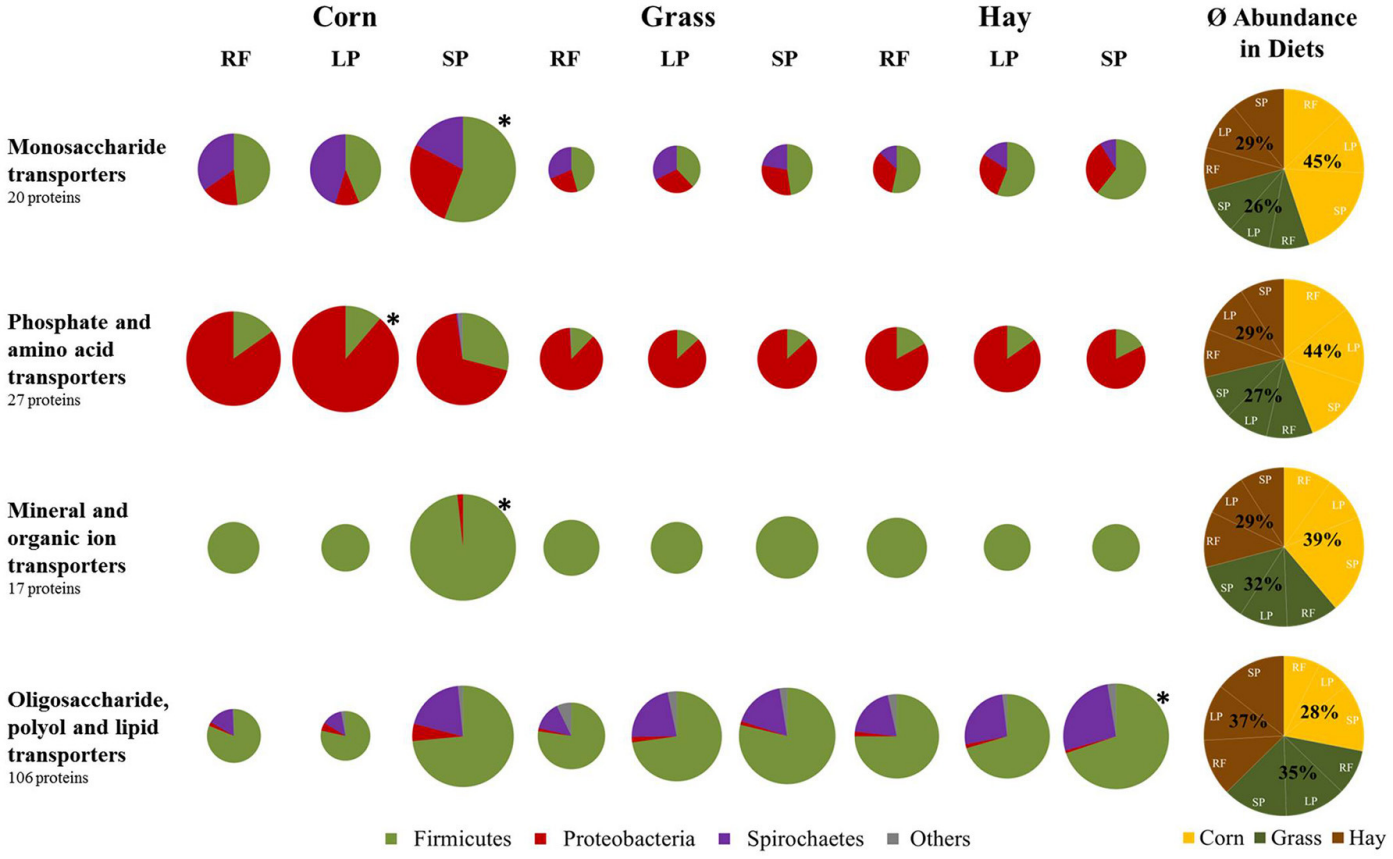
Varying abundance of bacterial ABC transporters in diets and sample fractions (*n* = 3). Pie sizes indicate the abundance in relation to the sample with the maximum LFQ-values (^*^) in each group. Phylogenetic origin of proteins in the respective samples is shown at phyla level. RF, rumen fluid; LP, liquid phase; SP, solid phase.

WebMGA assigned 7,745 and 336 KO identifiers to 8,163 bacterial and 358 archaeal proteins respectively (Table S4), revealing a total of 170 bacterial proteins with sequence similarity to ABC transporters (Table S5) as defined by the KO database. Figure 5 depicts the average abundance of the respective membrane transporters in diets and sample fractions in relation to the maximum LFQ-values within each group of transporters and the origin at bacterial phyla level. Analyses affirmed 106 proteins to the group of oligosaccharide, polyol, and lipid transporters that include several subunits of multiple sugar transport systems and of cellobiose, arabinose/lactose, maltose/maltodextrin, sorbitol/mannitol, and galactose oligomer transporters. The respective proteins were more abundant in the hay- and grass silage-based diets when compared to the corn silage-based diet (Figure 5). Within diets, the highest abundance was observed in the SP fractions. Oligosaccharide, polyol, and lipid transporters originated mainly from Firmicutes species. In contrast, the group of monosaccharide transporters, based on 20 proteins, was more abundant in the corn silage-based samples when compared to the grass silage- and hay-based diets (Figure 5). This group includes subunits of ribose, rhamnose, methyl-galactoside, and *sn*-glycerol 3-phosphate transport systems. Proteobacterial proteins showed an increased contribution regarding monosaccharide transporters when compared to the respective phylogenetic origin of the group of oligosaccharide, polyol, and lipid transporters (Figure 5). Phosphate and amino acid transporters based on 27 proteins showed the highest abundance in corn silage-derived samples and were mainly produced by Proteobacteria species (Figure 5). Mineral and organic ion transporters including 17 proteins were almost exclusively produced by Firmicutes species and showed the highest LFQ-values in the corn silage-derived SP fraction (Figure 5).

### Archaeal and bacterial enzymes involved in carbon metabolism

**Figure 6:**
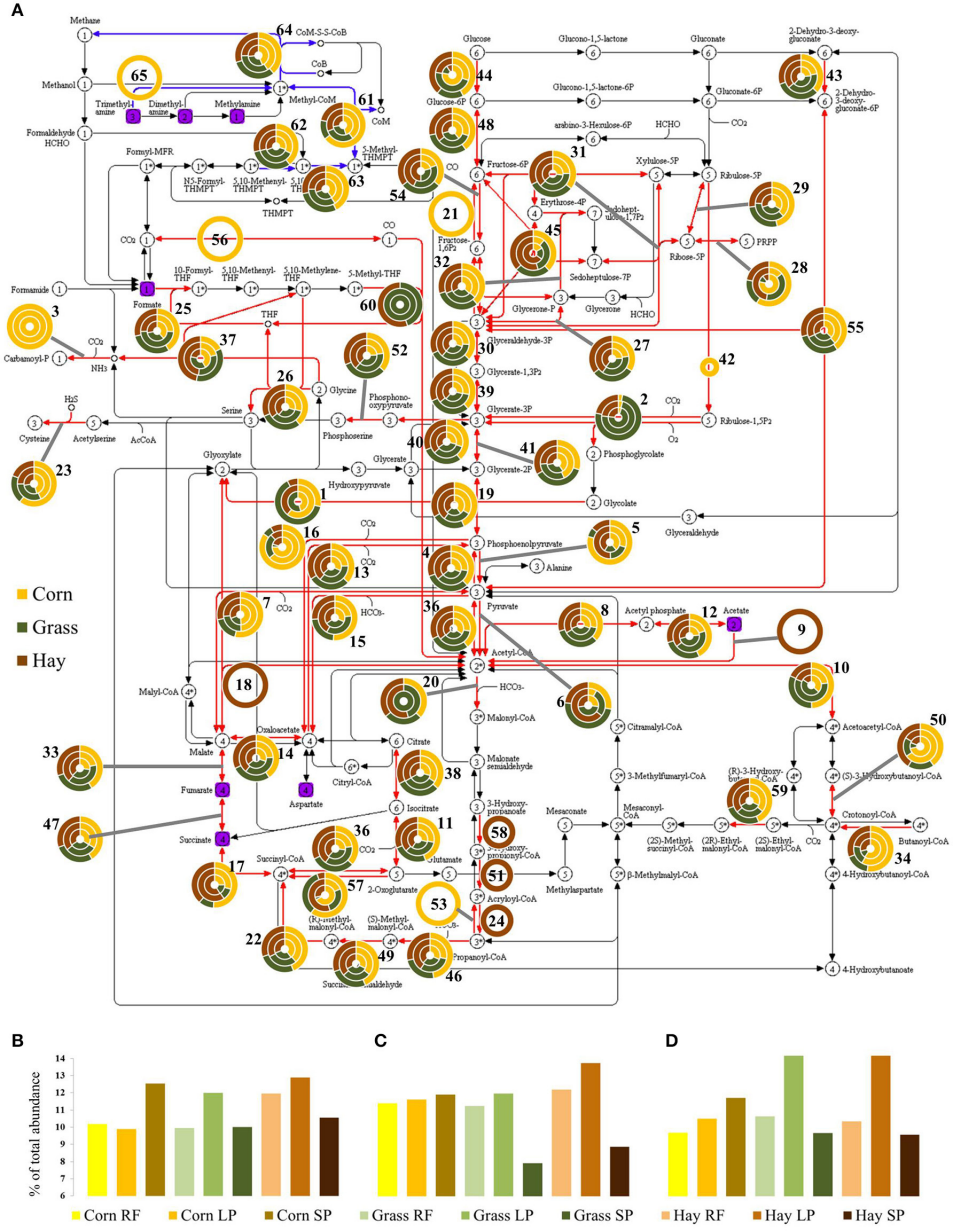
**(A)** Abundance of archaeal (blue) and bacterial (red) enzymes (*n* = 3) involved in carbon metabolism, grouped and numbered according to the respective reactions (Table S6). Outer circle, solid phase; middle circle, liquid phase; inner circle, rumen fluid. Purple, compounds identified by NMR. The percentage of total LFQ-values of groups involved in **(B)** the Embden-Meyerhof-Parnas pathway (5, 19, 27, 30, 32, 39, 41, 44, 48, 54), **(C)** citrate cycle (11, 14, 17, 33, 38, 47, 57) and **(D)** pentose phosphate pathway (29, 31, 45, 48). RF, rumen fluid; LP, liquid phase; SP, solid phase.

Varying abundance, in diets and sample fractions, of enzymes involved in the carbon metabolism are shown in Figure 6A. To visualize the abundance of the respective enzymes in the pathway map, a total of 1121 bacterial proteins assigned to 80 KO identifiers carrying out 70 KEGG REACTIONS were arranged into 60 functional groups Table S6). A few KOs were assigned to more than one KEGG REACTION and thus, appear more than once in the grouping. Furthermore, 28 archaeal proteins of eight KOs involved in the methane metabolism were grouped to five KEGG REACTIONS (Table S6). Additionally, eight compounds identified by NMR are shown in Figure 6A. Twenty-four enzymes of the groups 8 and 12 involved in the phosphate acetyltransferase-acetate kinase pathway were present in all diets and sample fractions, whereas only one acetyl-CoA synthetase from an uncultured bacterium in group 9 was found in the SP fraction of the hay-based diet (Figure 6A). The abundance of seven proteins of group 6 carrying out the oxidation of pyruvate to acetyl-CoA was higher in the RF and SP fractions of the grass silage-based diet and higher in the LP fraction of the hay-based diet when comparing to the other fractions and diets. Conversion of acetyl-CoA to malonyl-CoA employing two proteins of group 20 produced by *Bacillus* and *Prevotella* species, exhibited abundance exclusively in the grass silage-derived RF fraction and regarding the corn silage-based diet, were present only in the SP fraction (Figure 6A). A Tenericutes-derived carbamate kinase in group 3 producing carbamoyl phosphate, a metabolite in nitrogen disposal through the urea cycle, was found exclusively in the fractions of the corn silage-based diet (Figure 6A). Summing up the LFQ-values of groups involved in the Embden-Meyerhof pathway (5, 19, 27, 30, 32, 39, 41, 44, 48, 54) consisting of a total of 417 proteins, the abundance within diets was the highest in the LP fractions of the grass silage- and hay-based diets and the corn silage-derived SP fraction (Figure 6B). The sum of abundance of groups belonging to the citrate cycle (11, 14, 17, 33, 38, 47, 57) based on a total of 76 proteins exhibited lower LFQ-values in the SP fractions of the grass silage- and hay-based diets when compared to the respective RF and LP fractions and the corn silage-based samples (Figure 6C). Looking at the groups 29, 31, 45, 48 of the pentose phosphate pathway based on 34 proteins, the sum of LFQ-values was higher in the LP fractions of the grass silage- and hay-based diets when compared to the remaining samples (Figure 6D).

### Enzymes of short-chain fatty acid production

**Figure 7:**
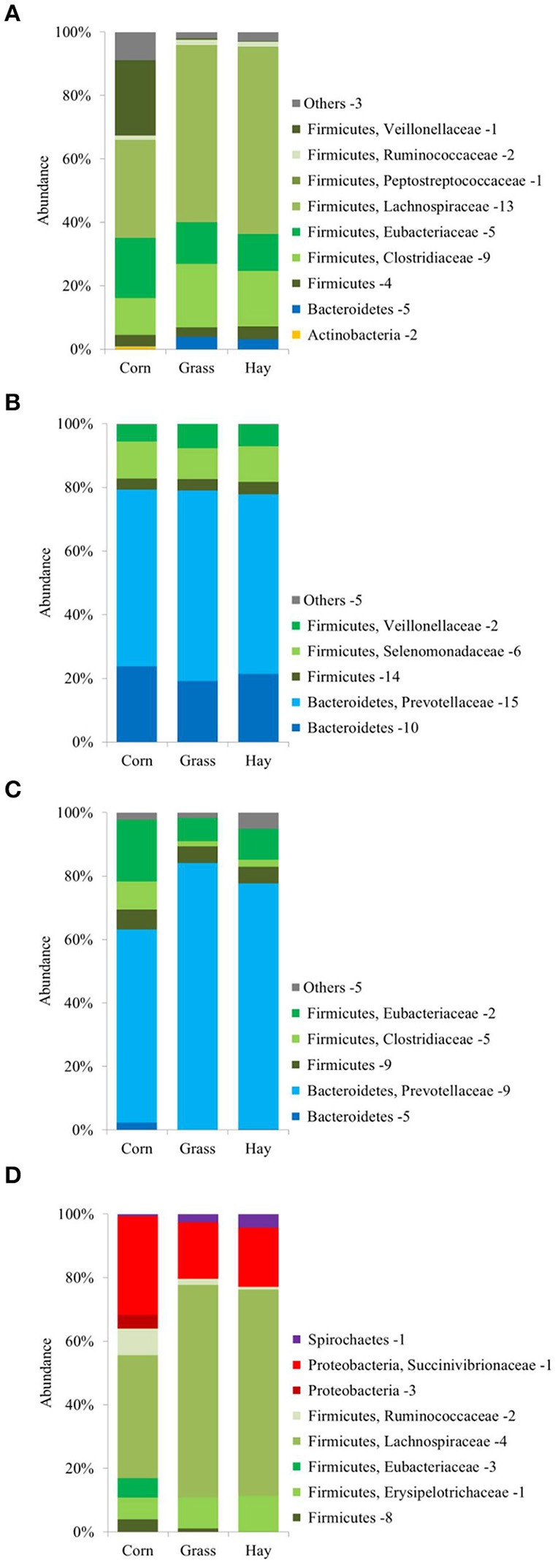
Average abundance and phylogenetic origin of bacterial enzymes involved in **(A)** butyrate, **(B)** propionate, **(C)** acetate, and **(D)** formic acid production in diets (*n* = 9). Numbers of assigned proteins are shown in the respective phylogenetic legends. For butyrate biosynthesis: COG4770 (acetyl/propionyl-CoA carboxylase), COG3426 (butyrate kinase), COG1250/COG1024 (3-hydroxyacyl-CoA dehydrogenase), COG0183 (acetyl-CoA acetyltransferase). For propionate biosynthesis: COG4799 (acetyl-CoA carboxylase), COG2185/COG1884 (methylmalonyl-CoA mutase). For acetate production: COG1012 (NAD-dependent aldehyde dehydrogenase), COG0282 (acetate kinase), COG0280 (phosphotransacetylase), and for formic acid COG1882 (formate acetyltransferase).

Bacterial enzymes involved in the production of acetate, butyrate, propionate and formic acid (Table S7) were retrieved using COG assignments of WebMGA (Table S4). The LFQ-values of proteins belonging to the respective COGs were summarized as described in Polansky et al. (2015). Figure 7 depicts the phylogenetic origin of the corresponding enzymes averaged over the diets. In total 45 enzymes involved in butyrate production were dominated by Firmicutes species constituting above 90% of the total abundance in all diets whereas enzymes from Actinobacteria appeared exclusively in the corn silage-based diet (Figure 7A). Five proteins of Bacteroidetes species showed a maximum of 3.9% of abundance in the grass silage-based samples and exhibited LFQ-values below 0.3% in the corn silage-based diet. Thirteen proteins of the Lachnospiraceae family exhibited the highest LFQ-values in the hay-based diet with 59% of the total abundance, decreasing in the grass (56%) and corn (31%) silage-based diets. Likewise, the abundance of two enzymes derived from Ruminococcaceae was higher in the grass silage- and hay-based diet (1.7%) when compared to the average in the corn silage-based diet (1.3%). In contrast, five enzymes of Eubacteriaceae showed the highest LFQ-values in the corn silage-based diet and decreased in the grass silage- and hay-derived samples. A 3-hydroxybutyryl-CoA dehydrogenase of *Megasphaera elsdenii* belonging to the family of Veillonellaceae was present in high abundance in the corn silage-based diet (24%) while the LFQ-values in the grass silage- and hay-based diets accounted for 0.4% and 0.1% respectively (Figure 7A). Propionate production based on 52 proteins was dominated by 25 enzymes from Bacteroidetes species, mainly Prevotellaceae, composing above 75% of the total abundance in all samples (Figure 7B). Correspondingly, nine enzymes of the Prevotellaceae family prevailed in acetate production constituting above 60% of abundance in the corn silage-based diet increasing to 77% in the hay-based diet and 84% in the grass silage-based diet (Figure 7C). Two enzymes of *Eubacterium* species showed an abundance of 20% while decreasing in the hay- and the grass silage-based diets with 10 and 7% respectively. Concerning the 23 enzymes involved in formic acid production, four proteins from Lachnospiraceae species constituted 39% of abundance in the corn silage-based diet whereas reaching 65% in the hay- and 67% in the grass silage-derived samples (Figure 7D). A formate C-acetyltransferase of *Succinatimonas hippie* belonging to the family of Succinivibrionaceae reached 31% of the total abundance in the corn silage-based diet and decreased to 19% in the hay- and 18% in the grass silage-based diet. Three formate acetyltransferases of *Eubacterium* species showed the maximum abundance of 6% in the corn silage-derived samples and were not present in the grass silage- and hay-based diets (Figure 7).

### Metabolites in rumen fluid

NMR spectroscopy allowed the quantification of 20 different compounds in all RF samples. Two-dimensional NMR spectroscopy further validated the presence of 12 compounds (^*^) including the major short-chain fatty acids acetate, butyrate, propionate, and valerate (Table 3). There were no statistically significant alterations of the metabolites regarding the different diets as determined by one-way ANOVA (*P* > 0.05). Overall, acetate was detected in highest concentrations ranging from 54.86 mM in the corn silage-based samples to 59.70 mM in the grass silage-derived RF samples. Propionate concentration was lower in the RF samples of the hay-based diet (13.86 mM) when compared to 15.22 and 15.94 mM in the corn silage- and grass silage-based samples, respectively (Table 3). The ratio of acetate to propionate concentration was highest in the hay-based samples and lowest in the corn silage-based samples. Butyrate was more abundant in the grass silage- and hay-derived RF samples (16.84 and 15.81 mM) when compared to 14.98 mM in the corn silage-based samples (Table 3). Valerate concentration was higher in corn silage- and hay-derived RF fractions (5.27 and 4.15 mM) when compared to 2.99 mM in the grass silage-based diet. The corn silage-based samples exhibited the highest concentration of succinate (0.38 mM) in comparison to the grass silage- and hay-based samples with 0.1 and 0.25 mM respectively (Table 3). Higher amounts of lactate were found in in the hay diet-based samples (5.25 mM) when compared to the corn silage- (1.18 mM) and grass silage-based samples (0.84 mM). Methylamine, dimethylamine, and trimethylamine were less abundant in the corn silage-based samples when compared to the grass silage and hay-based RF fractions (Table 3). Several compounds identified in low concentrations like aspartate, formate, fumarate, methylamines, and succinate are involved in the carbon metabolism as indicated in Figure 6.

**Table 3:** Average (∅) concentration (mM) and standard error of mean (SEM) of compounds identified in the RF fractions (*n* = 3) are shown.

**Metabolite**	**Average concentration (mM) and SEM (*n* = 3)**					
	**Corn RF**		**Grass RF**		**Hay RF**	
	**∅**	**SEM**	**∅**	**SEM**	**∅**	**SEM**
2-Phenylpropionate^*^	0.14	0.02	0.12	0.05	0.15	0.05
3-Phenylpropionate^*^	0.38	0.05	0.40	0.08	0.39	0.10
Acetate^*^	54.86	3.79	59.70	8.49	55.95	3.41
Adipate	1.78	0.67	1.66	0.34	1.45	0.58
Aspartate	0.23	0.07	0.39	0.15	0.31	0.12
Butyrate^*^	14.98	2.20	16.84	5.58	15.81	1.54
Dimethylamine^*^	0.10	0.07	0.29	0.08	0.28	0.05
Formate	0.14	0.03	0.10	0.02	0.11	0.02
Fumarate	0.02	0.00	0.01	0.00	0.02	0.00
Isobutyrate^*^	1.43	0.57	1.29	0.42	1.51	0.49
Isovalerate^*^	1.31	0.26	1.09	0.32	1.02	0.04
Lactate	1.18	1.11	0.84	0.45	5.25	4.35
Methylamine^*^	0.49	0.15	0.94	0.12	0.70	0.16
Phenylacetate^*^	0.13	0.05	0.17	0.11	0.19	0.09
Pimelate	4.54	0.53	3.13	0.70	3.73	0.19
Propionate^*^	15.22	3.17	15.94	3.34	13.86	1.30
Succinate	0.38	0.38	0.10	0.05	0.25	0.11
Trimethylamine^*^	0.17	0.13	0.59	0.35	0.62	0.49
Urea	2.74	1.21	2.68	1.54	2.98	0.86
Valerate^*^	5.27	0.95	2.99	0.42	4.15	0.31
Acetate : Propionate	3.60		3.75		4.04	

* *Compounds validated by 2D-NMR spectroscopy. RF, rumen fluid. There was no statistically significant difference in metabolite abundance between the diets (P > 0.05)*.

## Discussion

In this study, the dietary effects of the most common forages in cattle production on the structure and function of the archaeal and bacterial communities inhabiting different microenvironments of the rumen ecosystem were analyzed by a combination of shotgun-metaproteomics, Illumina amplicon sequencing and nuclear magnetic resonance to provide deeper insights into the complex microbial adaptation to varying substrates. In general, the bacterial community composition of the rumen samples was dominated by Bacteroidetes and Firmicutes species as reported by other nucleic acid-based studies (Jami and Mizrahi, 2012; Jami et al., 2014). However, the bacterial community arrangement changed significantly in response to varying diets and differed between the rumen sample fractions as confirmed by ANOSIM for both, the 16S rRNA gene and the metaproteomic analysis. There was no significant effect of the host animals on the inherent bacterial community structure (Table 2).

### Bacterial community composition and activity is influenced by dietary treatments

Dietary impact is the main factor shaping bacterial communities in the rumen (Ley et al., 2008; Henderson et al., 2015). The higher amount of non-fiber carbohydrates, mainly starch and sugars, in the corn silage-based diet (Table 1) might be responsible for the increased LFQ and OTU abundances of members of the Proteobacteria phylum and the family of Succinivibrionaceae (Hespell, 1992; Bryant, 2015). Typical members of the Succinivibrionaceae family are *Succinimonas amylolytica* and *Ruminobacter amylophilus*. The latter one is restricted to starch and maltose as fermentation substrates and produces succinic, formic and acetic acids (Hamlin and Hungate, 1956). *S. amylolytica* was shown to increase in abundance when substrates contained starch with succinic, acetic and propionic acid being the main fermentation products (Bryant et al., 1958). Thus, the higher amount of non-fiber carbohydrates in the corn silage-based diet (Table 1) and the associated rise in abundance of Proteobacteria and Succinivibrionaceae species (Figure 2) might have caused an increased production of succinate. The NMR-based metabolomic analysis determined the highest concentration of succinate in the corn silage-based RF fractions (Table 3). Moreover, the rise of Proteobacteria in the fractions of the corn silage-based diet (Figure 2) is in concordance with the increased abundance of monosaccharide transporters in the respective samples and the higher proteobacterial contribution to monosaccharide transporters when compared to the group of oligosaccharide transporters (Figure 5). Additionally, the abundance of proteobacterial glycosyltransferases and particularly carbohydrate esterases increased in the corn silage-derived LP and SP fractions (Figure 4).

**Figure 8:**
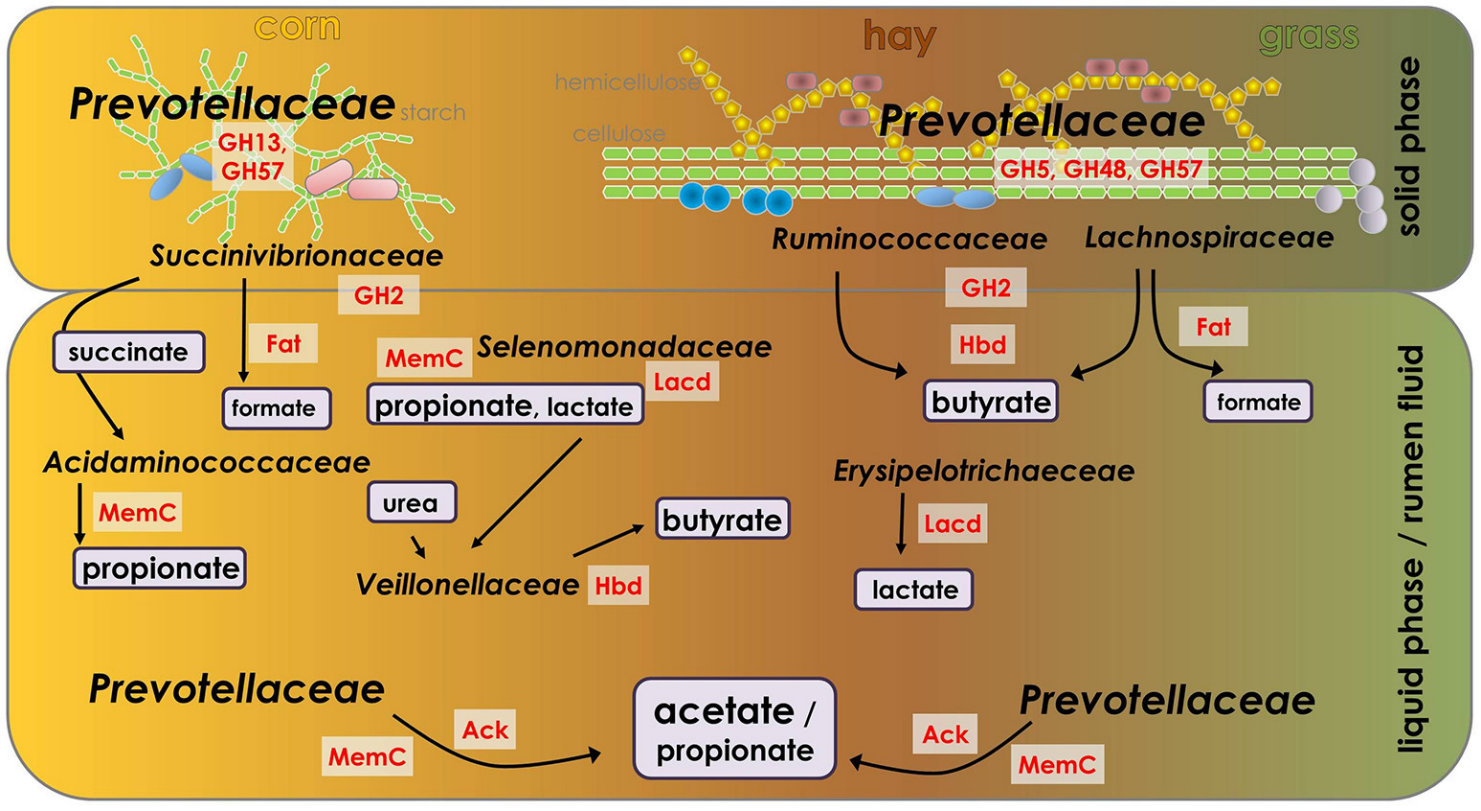
Consolidation of metaproteomic, 16S rDNA sequencing and metabolomic analyses showing the most active and abundant rumen bacteria in the respective diets and sample fractions and their main fermentation products. Character size of bacterial family names and metabolites are in accordance to their abundance (LFQ for proteins, concentration for metabolites). Ack, acetate kinase; Fat, formate acetyltransferase; GH, glycosyl hydrolase; Hbd, hydroxybutyryl-CoA dehydrogenase; Lacd, lactate dehydrogenase; MemC methylmalonyl-CoA mutase.

Beyond, the higher abundance of the Proteobacteria phylum and the Succinivibrionaceae family in the corn silage-based samples and the therewith associated increase in succinate formation might explain the rise of OTUs and proteins of the Acidaminococcaceae family in the samples of the corn silage-based diet (Figure 8). All OTUs of the Acidaminococcaceae family were assigned to the genus of the succinate-fermenting *Succiniclasticum* (van Gylswyk, 1995) and 48 of 70 identified proteins were produced by the asaccharolytic and succinate-utilizing *Phascolarctobacterium* species (Watanabe et al., 2012). *Succiniclasticum* and *Phascolarctobacterium* species of the Acidaminococcaceae family ferment succinate to produce propionate (Figure 8), the most important carbon source for the ruminant's gluconeogenesis (Yost et al., 1977). In total six proteins of *Phascolarctobacterium* species involved in propionate production were identified (Table S7). There are reports about reduced methane emissions under corn silage-based dietary regimen (Beauchemin and McGinn, 2005; van Gastelen et al., 2015). A metagenomics study linked a decrease in methane emissions and abundance of methanogenic archaea to an increased abundance of the Succinivibrionaceae family (Wallace et al., 2015) whose members utilize hydrogen to produce succinate which is then rapidly converted to propionate and in this compete with the most common, hydrogenotrophic methanogenesis (Liu and Whitman, 2008; McCabe et al., 2015). Concerning the present study, the ratio of acetate to propionate, an indicator for methanogenic activity, was lowest in the corn silage-based diet (Table 3). Furthermore, the increased abundance levels of the Succinivibrionaceae family (Figure 2), the higher amount of succinate (Table 3) and the increased abundance of six propionate-producing *Phascolarctobacterium* proteins in the fractions of the corn silage-based diet may indicate a consistency with the above-mentioned investigations.

No OTUs were assigned to the Firmicutes order of Bacillales or the family of Selenomonadaceae with its typical members of the rumen ecosystem, *Selenomonas ruminantium* and *Anaerovibrio lipolytica*. In contrast, 219 proteins were assigned to the family of Selenomonadaceae with the majority belonging to saccharolytic *Selenomonas* and lipolytic *Anaerovibrio* species, respectively. This may emphasize the benefits and the necessity of applying multiple, complementary methods to investigate the microbiomes of complex ecosystems like the rumen. A previous study analyzed the identical sample material by quantitative real-time PCR and reported no dietary impact on the abundance of *S. ruminantium* (Lengowski et al., 2016). Contrarily, the LFQ-values of proteins of the Selenomonadaceae family assessed in the present study revealed a lower abundance throughout the grass silage-based diet when compared to the corn silage- and the hay-based samples (Figure 2) including proteins involved in propionate formation (Table S7) and with sequence similarity to SLH (Table S3). The Gram-negative staining but phylogenetically Gram-positive rumen anaerobe *S. ruminantium* exhibits peptidoglycan-associated proteins with SLH that play an important role in the maintenance of the cell surface structure (Kojima et al., 2010). Furthermore, *S. ruminantium* species are characterized by their ability to use a broad range of substrates including the fermentation products of other bacteria (Bryant, 1956; Cotta, 1990; Rasmussen, 1993). Cross-feeding between *S. ruminantium* and *Butyrivibrio fibrisolvens* was reported before (Cotta and Zeltwanger, 1995). Another study showed that the co-cultivation of *S. ruminantium* and *B. fibrisolvens* promoted the growth of *S. ruminantium* (Cotta, 1992). Correspondingly, the LFQ-values of 124 *Butyrivibrio* proteins assessed by metaproteomics exhibited the lowest abundance in the samples of the grass silage-based diet. Thus, the lower abundance of the Selenomonadaceae family in the grass silage-based diet might be linked to a similarly decreased abundance of the *Butyrivibrio* proteins in the respective samples.

The family of Erysipelotrichaceae of the Clostridium subphylum cluster XVII as well belonging to the phylum of Firmicutes exhibited the highest abundance of LFQs and particularly OTUs in the sample fractions of the hay-based diet (Figure 2). Most of the respective OTUs were assigned to the genera of *Sharpea* and *Kandleria* while most proteins were produced by species belonging to the genera of *Coprobacillus, Catenibacterium*, and *Eggerthia*. Due to phenotypic, chemotaxonomic and phylogenetic data, it was suggested that *Lactobacillus catenaformis* and *L. vitulinus* should be reclassified into the genera of *Eggerthia* and *Kandleria*, respectively (Salvetti et al., 2011). Similar to *Lactobacillus* species, most members of Erysipelotrichaceae family probably ferment a wide range of sugars to produce mainly lactic acid (Figure 8) as reported for *Sharpea azabuensis* (Morita et al., 2008). This assumption may be supported by the higher amounts of lactate identified in the RF samples of the hay-based diet (Table 3). Moreover, studies of the sheep rumen linked a higher abundance of the Erysipelotrichaceae family in low-methane emitting animals to an increased lactic acid production in which less hydrogen and thus less methane is formed (Kittelmann et al., 2014; Kamke et al., 2016). The higher abundance of the Erysipelotrichaceae family, mainly *Sharpea* species was further associated with an increased abundance of *Megasphaera* species that convert formed lactate to butyrate (Kamke et al., 2016). In contrast, the higher abundance of the Erysipelotrichaceae family in samples of the hay-based diet determined in the present study was not accompanied by an increased abundance of *Megasphaera* species or the Veillonellaceae family that exhibited the highest abundance in the corn silage-based samples (Table S2). Fernando et al. (2010) reported a similar increase of *Megasphaera* species upon high grain diets. It was hypothesized that the enrichment of the Erysipelotrichaceae family is related to the smaller rumen size and higher ruminal turnover rates of low-methane emitting sheep which favors microorganisms with fast, hetero-fermentative growth on sugars (Kittelmann et al., 2014; Kamke et al., 2016). However, in the present study the rise of the Erysipelotrichaceae family in the larger rumen of the Jersey cows was observed exclusively under hay-based dietary regimen and the ratio of acetate to propionate, an indicator for methanogenic activity was highest in the hay-based diet (Table 3). The findings of the present study suggest further microbiological investigations of the yet sparsely described family of Erysipelotrichaceae to obtain a more valid basis regarding their functional capabilities and their role within the rumen ecosystem.

Bifidobacterial genomes comprise features necessary for the metabolism of plant-derived complex carbohydrates like glycoside hydrolases and sugar ABC transporters (Pokusaeva et al., 2011). This might explain the higher abundance of proteins and OTUs of the family of Bifidobacteriaceae in the more fiber-rich grass silage- and hay-based samples when compared to the corn silage-based diet (Table S2). Moreover, a *Bifidobacterium*-derived glycosyltransferase of the family GT4 and oligosaccharide, polyol, and lipid transporters from Bifidobacteriaceae were more abundant in the grass silage-based diet and the hay-based samples when compared to the relatively low abundance in the corn-silage based diet. Furthermore, the polysaccharide-degrading capabilities of Actinobacteria including the Bifidobacteriaceae family might be reflected by the higher abundance in the SP fractions of all diets (Figure 2). Contrarily, the study of de Menezes et al. (2011) reported a higher abundance of 16S rRNA gene sequences of Actinobacteria in the LP fractions.

### Bacterial community composition and activity is influenced by the microenvironment

Besides the effect of animal feed composition, large variations in community structure between the different microenvironments of the rumen ecosystem were observed emphasizing the importance of sample fractionation in rumen studies to cover the effects of treatments throughout the whole ecosystem and its specific functional niches. The difference in bacterial community composition between sample fractions was shown to be greater than the difference between the same fractions of individual animals (Kong et al., 2010). Variations between the different microenvironments have already been discovered before the advent of 16S rRNA gene-based community analysis since the chemical composition of firmly-attached bacteria was shown to be different from the liquid-associated population whereas loosely-attached bacteria were rather similar to the liquid-associated population (Legay-Carmier and Bauchart, 1989) which confirms the findings of the present study. Within diets, the more fiber-rich SP fractions revealed a significant increase in abundance of polysaccharide degrading species of the Firmicutes order of Clostridiales including the families of Lachnospiraceae and Ruminococcaceae as found by OTU and protein abundance levels (Figure 2). The order of Clostridiales includes several cellulolytic *Clostridium* species (Vos et al., 2011) and as well fiber-degrading members of the Eubacteriaceae family (Prins et al., 1972; Van Gylswyk and Van der Toorn, 1985). Most of the OTUs within the order of Clostridiales, excepting Lachnospiraceae and Ruminococcaceae, were assigned to unclassified Clostridiales but no OTUs were assigned to the family of Eubacteriaceae while most respective proteins were produced by Eubacterium and Clostridium species. The family of Lachnospiraceae comprises the prominent cellulolytic *B. fibrisolvens* (Bryant and Small, 1956) and the pectinolytic *Lachnospira multiparus* (Duskova and Marounek, 2001). Members of the Lachnospiraceae family exhibit strong hydrolyzing activities with multiple sets of carbohydrate-active enzymes (Stackebrandt, 2014) which may explain the increased abundance in the SP fractions of all diets (Figure 2). The group of oligosaccharide, polyol and lipid transporters contained 27 Lachnospiraceae proteins that exhibited the highest abundance in the SP fractions of all diets (43%) when compared to the LP (29%) and the RF (28%) fractions. A similar increase in abundance of Lachnospiraceae species in the fiber-adherent fractions was reported by Larue et al. (2005). Butyrate formation seemed to be performed by the members of the Lachnospiraceae in the two fiber-rich diets (Figure 8) as proteins involved in butyrate formation revealed the highest abundance (Figure 7) and butyrate concentration was higher in the RF fractions of these samples (Table 3). Comparably, protein and OTU abundances of the Ruminococcaceae family with its prominent cellulolytic representatives *Ruminococcus flavefaciens* and *Ruminococcus albus* were not significantly influenced by the dietary treatments but the respective proteins were more abundant in the SP fraction of all diets. A comprehensive study of Henderson et al. (2015) reported a relatively even distribution of *Ruminococcus* species across different diets and host animals comparable to the finding of the present study. The presence of phenylpropionate identified by NMR (Table 3) was reported to be essential for adherence to and degradation of cellulose by *R. albus* (Stack and Hungate, 1984). The phylum of Fibrobacteres is represented by the major cellulose degrader *Fibrobacter succinogenes* that is restricted to cellulose, hemicellulose or glucose as growth substrates (Hungate, 1950; Puniya et al., 2015). However, there were no significant dietary effects on the abundance of Fibrobacteres proteins and OTUs but within diets, the LFQ-values reached their maximum in the SP fractions (Figure 2) similar to the findings of a previous DNA sequencing study (de Menezes et al., 2011). Large differences between protein- and the OTU-based abundances might be explained by the fact that metaproteomic investigations depend on the amount and quality of reference sequences available for database searches (Seifert et al., 2013; Tanca et al., 2013). Currently only 15 genomes and thus, comparably small numbers of annotated protein sequences are available for the phylum of Fibrobacteres which limits mass spectrometry-based identifications. In contrast, 1,863 different 16S rRNA gene sequences of the phylum of Fibrobacteres are deposited in the RDP database.

The phylum of Spirochaetes was more abundant in the SP fractions as determined by both methods (Figure 2) confirming the findings of de Menezes et al. (2011). The majority of proteins and OTUs belonged to *Treponema* species. *Treponema bryantii* is known to interact with cellulolytic bacteria (Stanton and Canale-Parola, 1980) and *T. succinifaciens* is able to ferment carbohydrates (Cwyk and Canale-Parola, 1979) which may explain the increased abundance throughout the SP fractions. Comparably, the low abundant phylum of Synergistetes, characterized in 2009, revealed the highest LFQ-values of proteins and particularly OTUs in the SP fractions (Table S2). There is not much information about the members of this phylum that are present in many anaerobic ecosystems including the gastrointestinal tract of animals but usually appear in low abundance within the respective environments (Jumas-Bilak and Marchandin, 2014). *Synergistes jonesii* was first isolated from the rumen of goat and did not ferment carbohydrates but is thought to be involved in the degradation of plant-derived toxins such as mimosine and thus, might be beneficial for the host animal (Allison et al., 1992). The overall low abundant phyla of Elusimicrobia and Verrucomicrobia were not affected by the different diets or sample fractions. However, the abundance of Elusimicrobia proteins was lower when compared to the respective OTUs whereas the abundance of Verrucomicrobia proteins was higher when compared to the corresponding OTUs (Table S2). A study employing total RNA sequencing as well as targeted RNA- and DNA amplicon sequencing identified the phyla of Elusimicrobia and Verrucomicrobia exclusively in the RNA-based datasets and proposed a higher activity of the respective phyla in the rumen (Li et al., 2016). The present data may confirm a high metabolic activity of the Verrucomicrobia phylum due to the higher abundance of proteins when compared to the respective OTUs.

The Prevotellaceae family comprises common rumen bacteria such as *Prevotella ruminicola, P. brevis, P. bryantii*, and *P. albensis* and was shown to increase in abundance upon inclusion of concentrate in diets (Henderson et al., 2015). In the present study, the Prevotellaceae family constituted the most dominant bacterial family within the rumen ecosystem as reported before (Kim et al., 2011; Jami and Mizrahi, 2012). The abundance of proteins and OTUs assigned to the Prevotellaceae family was not affected by the different diets but within diets, the LFQ-values were highest in all LP fractions (Figure 2). A higher abundance of the Prevotellaceae family in the liquid rumen fractions has been reported before (Whitford et al., 1998; Kocherginskaya et al., 2001; Pitta et al., 2010). Members of the Prevotellaceae family are characterized by their versatile metabolic capabilities and their ability to utilize a broad range of substrates including peptides, proteins, monosaccharides, and plant polysaccharides (Miyazaki et al., 1997; Matsui et al., 2000; Purushe et al., 2010) and thus, may not be primarily affected by changes in diet composition. The majority of identified CAZymes were produced by Prevotellaceae species including 46 glycoside hydrolases, 14 glycosyltransferases and six carbohydrate esterases emphasizing their functional prevalence (Table S3). Furthermore, most enzymes involved in acetate and propionate formation were derived from Prevotellaceae species (Figure 8).

The high abundance of polysaccharide-degrading bacteria in the SP fractions described above might have caused an increased availability of monosaccharides and a higher abundance of proteins involved in glycolysis by the Embden-Meyerhof-Parnas pathway. Considering the different texture and the higher amounts of starch in the corn silage-based diet when compared to the more fibrous grass silage- and hay-based diets most monosaccharides possibly were present in the SP fraction of the corn silage-based diet whereas most monosaccharides from the degradation of structural plant polysaccharides were rather present in the LP fractions of the grass silage- and hay-based diets (Figure 6B). Similar to reports of Pitta et al. (2010), more fibrous hay diets included the development of a digesta mat with clearly separated phases whereas wheat-based rumen content was more homogenized without a distinct fibrous mat. Proteins involved in the citric acid cycle showed a remarkably low abundance in the SP fractions of the grass silage- and hay-based diets (Figure 6C) pointing toward a low abundance of substrates like succinate which is in accordance with the abundance values of proteins of the Embden-Meyerhof-Parnas pathway. This might explain as well the high abundance of the pentose phosphate pathway in the LP fractions of the grass silage- and hay-based diets that probably contained most sugars within the fiber-rich diets (Figure 6D).

Most studies of the rumen metabolome identified higher numbers of different metabolites by a combination of NMR- and more sensitive MS-based approaches (Ametaj et al., 2010; Saleem et al., 2012, 2013). However, despite the statistical insignificancies the metabolite concentrations assessed by NMR in the present study further supported the findings of the 16S rRNA gene sequencing and the metaproteomic analysis. Insignificant metabolite patterns might also be related to the 52% identical composition of the experimental diets used in the present study.

### Archaeal community differs in sequence and protein composition

There were no diet, sample fraction or host related shifts in community structure of archaea probably due to the less versatile metabolic capabilities when compared to bacteria (Henderson et al., 2015) and the relatively low numbers of identified proteins and OTUs. The findings of the present study support the results of a previous study that analyzed the abundance of total methanogens and the Rumen Cluster C in the same sample material using quantitative real-time PCR (Lengowski et al., 2016). However, in contrast to the bacterial datasets, the archaeal community composition inferred from the quantified proteins differed clearly from the structure obtained by Illumina amplicon sequencing (Figure 3). OTUs were exclusively assigned to three families of methanogens with members of the family Methanobacteriaceae and Methanomassiliicoccaceae being dominant throughout all diets and sample fractions similar to the results of other 16S rRNA gene-based studies (Janssen and Kirs, 2008; Seedorf et al., 2015). On the other hand, the phylogenetic composition inferred from the quantified archaeal proteins depicted a higher diversity including the presence of the Crenarchaeota and Thaumarchaeota phyla that were identified in low abundance in the rumen ecosystem before (Shin et al., 2004; Wang et al., 2016; Jin et al., 2017). The different phylogenetic distributions of archaeal proteins and OTUs might be attributed to the prevalence of *Methanobrevibacter ruminantium* and *M. gottschalkii* of the Methanobacteriaceae family which dominate the ruminal archaeal community (Janssen and Kirs, 2008; St-Pierre and Wright, 2013; Seedorf et al., 2015; Danielsson et al., 2017). High numbers of the respective 16S rRNA genes might have prevented sufficient sequencing reads of low abundant 16S rRNA genes in the present study. Moreover, some archaea are endo- and ectosymbiotically linked to protozoa (Lange et al., 2005). In particular species of Methanobacteriaceae family were found to be associated with protozoa (Janssen and Kirs, 2008). The specific sample preparation protocols for the shotgun metaproteomic analysis focusing on the enrichment of prokaryotic cells might have caused bias against protozoa-associated archaea, which could further explain the differences between the DNA- and protein-derived datasets. The metaproteomic approach identified the alpha, beta and gamma subunits of the methyl-coenzyme M reductase based on 15 proteins (Table S7) that constituted 23% of the total abundance of 55 proteins that belonged to the methane metabolism as defined by the KEGG database. Eleven proteins were produced by species of the Methanobacteriaceae family and four proteins were assigned to the Thermoplasmata phylum. The methyl-coenzyme M reductase catalyzes the final step in the formation of methane (Ragsdale, 2014) and the mcrA gene, encoding the alpha subunit of the methyl-coenzyme M reductase, is preferably used as a functional marker since present in all methanogens (Denman et al., 2007). The abundance of the subunits of the methyl-coenzyme M reductase (Table S1) was lowest in the corn silage-based diet corresponding to the ratio of acetate to propionate that was lowest in the corn silage-based diet as well (Table 3).

Next to the hydrogenotrophic methanogenesis, methylotrophic methanogens like the family of Methanomassiliicoccaceae belonging to the class of Thermoplasmata utilize compounds like methylamine, dimethylamine, and trimethylamine as their major energy and carbon sources. Trimethylamines are formed by bacteria from plant-derived glycine betaine and cholin (Neill et al., 1978; Mitchell et al., 1979). A trimethylamine corrinoid protein MttC of the methanogenic *archaeon ISO4-H5* was identified in the metaproteomic analysis and the NMR-based analysis found higher concentrations of methylamine, dimethylamine and trimethylamine in the grass silage- and hay-based diets (Table 3) which is supported by other reports that indicate higher glycine betaine concentrations in grass as when compared to corn (Lerma et al., 1991; Chendrimada et al., 2002). The ratio of acetate to propionate was higher in the two fiber-rich diets that further contained more than twice as much methylamines when compared to the corn silage-based diet suggesting a potentially higher activity of methylotrophic methanogenesis in the fiber-rich diets. A previous study suggested to focus on methylamines rather than on hydrogen for mitigating methane emission from the rumen (Poulsen et al., 2013).

## Conclusion

The obtained datasets revealed significant alterations of the structure and function of the microbial communities in response to the dietary treatments as determined unanimously by the protein- and the DNA-based analyses. Certain contrasts between the methods employed clearly emphasized the benefits of using combinations of complementary methods to study the microbiome of complex ecosystems like the rumen. Moreover, tremendous variations in community composition and functional patterns regarding the different microenvironments within the rumen were observed by both methods prompting for the necessity of sample fractionation in rumen studies to cover the effects of applied treatments throughout the whole ecosystem. The role of low abundant phyla such as Elusimicrobia, Synergistetes, Tenericutes, and Verrucomicrobia within the rumen ecosystems remains comparably vague since not much information about the respective species is available. This suggests microbiological investigations of the respective species in pure and mixed cultures to obtain more accurate data regarding their functional capabilities. This study may provide deeper insights into the complicated network of bacterial interactions and adaptions to various substrates. Bioinformatic and technical progress may enhance the metaproteomic coverage of future studies.

## Author contributions

The authors' contributions are as follows: MR and JS designed the research. SD, AC, JC, UB, MR, and JS conducted the research. SD, AC, JC, and JS analyzed the data. SD and JS wrote the manuscript. All authors read and approved the final version of the manuscript.

### Conflict of interest statement

The authors declare that the research was conducted in the absence of any commercial or financial relationships that could be construed as a potential conflict of interest.

## Supplementary material

Table S1 List of identified proteins and OTUs including the LFQ-values determined by MaxQuant.

Table S2 Percentage abundance of archaeal and bacterial proteins and OTUs at phyla, class, order, and family level.

Table S3 dbCAN assignments. List of identified CAZymes.

Table S4 WebMGA assignments. List of KEGG Orthology (KO) identifiers and Cluster of Orthologous Groups of proteins (COG) assigned to archaeal and bacterial proteins.

Table S5 List of ABC transporters.

Table S6 KEGG REACTIONS in carbon metabolism.

Table S7 List of enzymes involved in short-chain fatty acid production.
